# Control of switching between metastable superconducting states in δ-MoN nanowires

**DOI:** 10.1038/ncomms10250

**Published:** 2015-12-21

**Authors:** Jože Buh, Viktor Kabanov, Vladimir Baranov, Aleš Mrzel, Andrej Kovič, Dragan Mihailovic

**Affiliations:** 1 Department of Complex Matter, Jozef Stefan Institute, Jamova 39, 1000 Ljubljana, Slovenia; 2 Department of Physics, University of Antwerp, Groenenborgerlaan 171, 2020 Antwerp, Belgium; 3 Jozef Stefan International Postgraduate School, Jamova 39, 1000 Ljubljana, Slovenia; 4 Faculty of Mathematics and Physics, University of Ljubljana, 1000 Ljubljana, Slovenia

## Abstract

The superconducting state in one-dimensional nanosystems is very delicate. While fluctuations of the phase of the superconducting wave function lead to the spontaneous decay of persistent supercurrents in thin superconducting wires and nanocircuits, discrete phase-slip fluctuations can also lead to more exotic phenomena, such as the appearance of metastable superconducting states in current-bearing wires. Here we show that switching between different metastable superconducting states in δ-MoN nanowires can be very effectively manipulated by introducing small amplitude electrical noise. Furthermore, we show that deterministic switching between metastable superconducting states with different numbers of phase-slip centres can be achieved in both directions with small electrical current pulse perturbations of appropriate polarity. The observed current-controlled bi-stability is in remarkable agreement with theoretically predicted trajectories of the system switching between different limit cycle solutions of a model one-dimensional superconductor.

## Abstract (web summary)

Fluctuations of the phase of the superconducting wave function in one-dimensional nanosystems can lead to the appearance of metastable superconducting states. Here, the authors show that it is possible to manipulate the switching between such states by means of a small electrical noise in δ-MoN nanowires.

## Graphical abstract

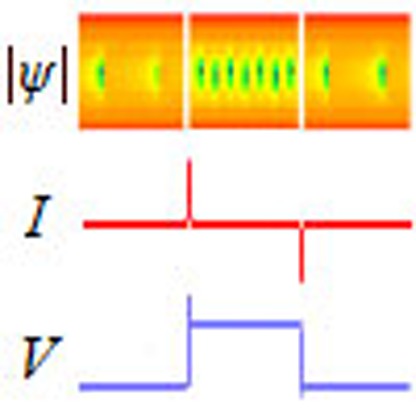

After the advent of superconductivity it was thought that in the bulk material the transition from the superconducting to the normal state in the presence of current occurs abruptly, as a result of superconducting state becoming unstable^1^,^2^. However, investigations^3^,^4^,^5^,^6^,^7^,^8^,^9^,^10^,^11^,^12^,^13^,^14^,^15^,^16^,^17^,^18^,^19^ of narrow superconducting channels revealed that very unusual dynamical resistance may appear in between the two states, whose properties are governed by spatial and temporal fluctuations of the phase *θ* of the complex-order parameter 
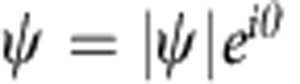
. Theory for such quasi-one-dimensional systems predicts a spatial variation of the order parameter of the form 
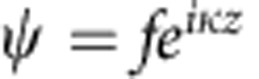
 where *f*=(1−*κ*^2^)^1/2^, and *κ* depends on the current density via 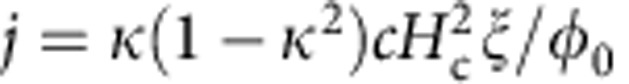 and *z* is distance along the wire measured in units of *ξ*. Here *H*_c_ is the thermodynamic critical field, *ξ* is the coherence length, and *φ*_0_=*πħc*/*e* is the flux quantum, *ħ* the reduced Planck's constant and *e* the electron charge. Although the realistic spatio-temporal behaviour in nanowires of finite dimensions is more complicated (and will be discussed later in the paper), the formula 
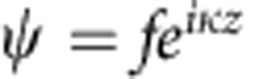
 qualitatively illustrates the behaviour of the order parameter in an ideal one-dimensional superconducting wire above the critical current (Fig. 1a). In accordance with the Josephson relation the voltage along the wire accelerates the superconducting electrons leading to an increase of *κ*. Since the voltage *V* is localized mainly near the phase-slip centre (PSC), *κ* will increase mainly in the vicinity of the PSC. According to the relation *f*=(1−*κ*^2^)^1/2^ the order parameter is strongly reduced near the PSC. Eventually, as it reaches zero, the phase slips by a multiple of 2*π* (Fig. 1a). After each phase-slip event the order parameter will recover and the process will repeat itself at the Josephson frequency *ν*_J_=*eV*/*h*. In the presence of a current *I*, each phase-slip event releases an energy *Iφ*_0_/*c*, at a rate *ν*_J_. Phase-slip process can also be triggered below the critical current either classically by thermal fluctuations^20^,^21^,^22^, or by quantum phase-slip (QPS) processes^23^,^24^ at low temperatures.

Such intrinsic phase-slip phenomena manifest themselves in two ways: (1) fluctuations induced PSCs lead to a finite resistivity of a superconducting wire, which depends on the number of PSCs^3^,^4^,^5^,^6^,^7^,^8^,^9^,^10^,^11^,^12^,^13^,^19^; and (2) current induced step-like current–voltage switching^5^,^14^,^15^,^16^,^25^,^26^. As a result of (1), theoretical modelling predicts that the resistivity of a superconducting wire is never zero, and depends exponentially on temperature^20^,^21^,^22^. Chen *et al*.^26^ recently showed spontaneous switching from a superconducting ground state below 650 mK to a non-equilibrium resistive state in Zn mediated by the so-called Andreev quasiparticles and demonstrating the coexistence of superconductivity with dissipation, while Astafiev *et al*.^19^ recently demonstrated a superconducting qubit based on QPS^27^.

Here we show that the switching between metastable superconducting states in δ-MoN nanowires can be externally controlled. Recent theoretical calculations^28^,^29^ imply that it might be possible to anticipate switching of the system between different limit cycles, corresponding to different solutions of the nonlinear time-dependent Ginzburg–Landau (TDGL) equations, by applying small external perturbations near bifurcation points (the ‘butterfly effect'). The conditions for observing controllable switching are somewhat delicate, because it can be easily inadvertently triggered by thermal fluctuations arising from Joule heating in the wire. To reduce this, one can go to low temperatures, but specific heat and thermal conductivity both limit to zero as *T*→0, so heating is an ever-increasing problem at low temperatures. On the other hand, if one reduces the current to avoid Joule heating then Schott noise or quantum fluctuations may trigger uncontrollable switching. The two conflicting requirements may be resolved with high-temperature operation, which requires a high superconducting critical temperature and good thermal management. Apart from this, we need a confined superconducting material with good control over lateral dimensions *d*.

## Results

### Description of the system

Recently, δ-MoN nanowires were synthesized from Mo_6_S_*y*_I_*z*_ (8.2<*y*+*z*<10) precursors^30^ (Supplementary Note 1). They appear to have a critical temperature (*T*_c_≃14 K), can be made with different diameters, and allow small Pt contacts to be fabricated with focused ion beam technology. A transmission electron micrograph of a cross-section of a single δ-MoN nanowire is shown in Supplementary Fig. 1. The material gives us an excellent opportunity to study fundamental phase-slip phenomena in thin nanowires with high *T*_c_, and, for the first time, direct external control. The details of the device circuit, fabrication and material characteristics are presented in the Methods section.

**Figure 1:**
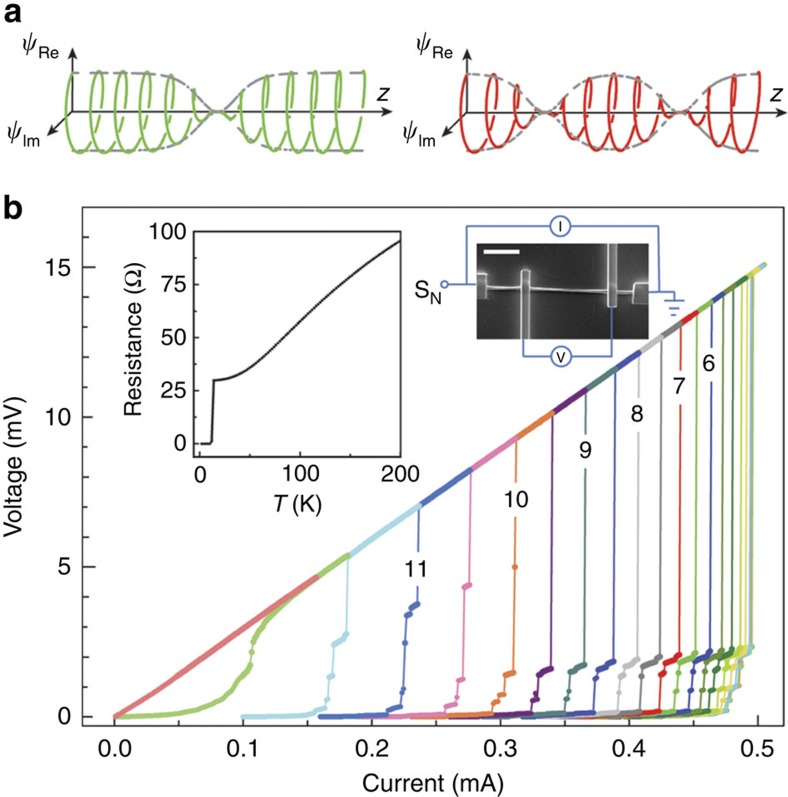
Switching between dissipative states. (**a**) Schematic illustrations of the spatial variation of the real and imaginary parts of 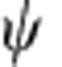 along the wire with one and two PSCs, respectively. (**b**) *V*–*I* curves for different temperatures between 2.5 and 12.5 K in 0.5 K steps (some values are indicated in the figure), measured with increasing current. Inset, resistance versus temperature and a scanning electron micrograph image of the measuring circuit with circuit diagram (white marker corresponds to 5 μm).

In the insets to Fig. 1b we show the δ-MoN nanowire circuit for a nanowire with *d*=320 nm with four contacts, and its resistance *R* as a function of temperature. (Thinner nanowires have a finite resistance down to lower temperatures, and show signs of QPS^7^,^9^,^10^,^25^, so they are not suitable for our purposes.) The voltage–current (*V*–*I*) characteristic of the wire at different temperatures between 2.5 and 12.5 K is shown in Fig. 1b. Multiple steps are observed, each step representing a change of the number of PSCs in the nanowire. The steps are more pronounced and sharper at lower temperatures.

**Figure 2:**
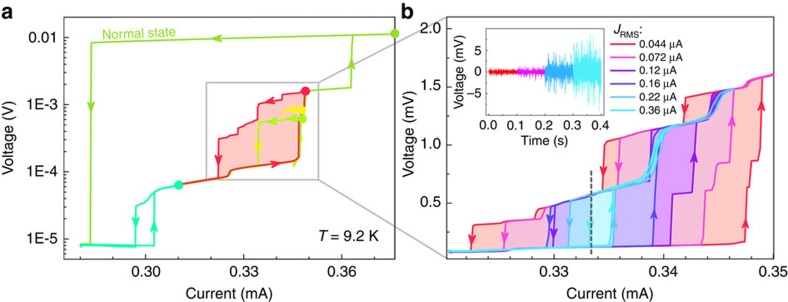
Hysteresis in *V*–*I* characteristics versus noise. (**a**) Current–voltage graphs at 9.2 K. Different hysteresis loops measured by ramping the source current to different values indicated by coloured circles. (**b**) The red-coloured loop from **a** measured with different r.m.s. noise levels (indicated) and shown in the inset. As the noise is increased the loop becomes narrower and smoother.

Cycling the current *I* exposes strongly hysteretic behaviour, with multiple intermediate states (Fig. 2a). The area of the hysteresis loops depends on the end-point value of *I* reached in each cycle. The largest loop is observed when the current is cycled up all the way to the normal state. In this case, on the return cycle, on reducing the current, the system eventually jumps directly from the normal state to the superconducting ground state with no intermediate states. The switching occurs on a timescale *ν*_J_, which is much shorter than the electrical time resolution of the present measuring system (∼1 μs).

### Noise switching

The application of an external noise source has a profound influence on the *V*–*I* curves. In Fig. 2b we show the data for the same sample measured with different levels of external noise, whose root mean squared (r.m.s.) values are given in the inset. (The noise characteristics are shown in Supplementary Fig. 2.) With increasing noise we see two effects: (1) the current needed to switch to a higher state is reduced such that the area enclosed by the hysteresis loop is reduced; and (2) the curves become smoother.

**Figure 3:**
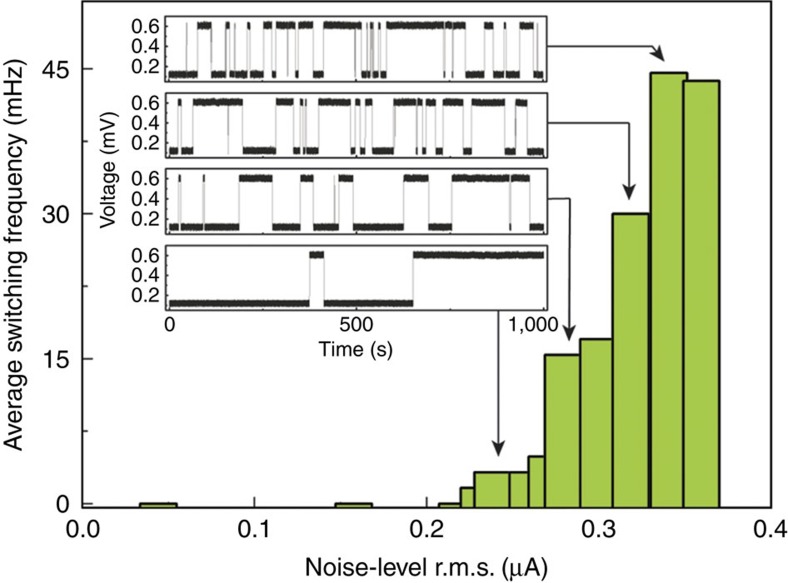
Noise control of telegraph switching frequency. At a fixed current of 0.3334, mA (indicated by grey dashed line in Fig. 2b) the amplitude of the noise is varied. The bar graph shows the average frequency of the switching at different noise r.m.s. levels. Several examples of the switching dynamics are shown in the inset.

By setting the bias current within the hysteresis loop, the voltage exhibits telegraph noise; pseudo-random jumps between two different states, of which one or both are dissipative (Supplementary Note 2). The current dependence of the telegraph noise dynamics is shown in Supplementary Fig. 3. We see that adding external noise provides a small perturbation to the system that enhances the system switching from one state to another. At a fixed current, the total time the system spends in each state is the same regardless of the level of the external noise. However, the frequency of switching between the two states is strongly dependent on the noise level, as shown in Fig. 3 for *I*=0.3334, mA at 9.2 K.

### Current pulse switching

**Figure 4:**
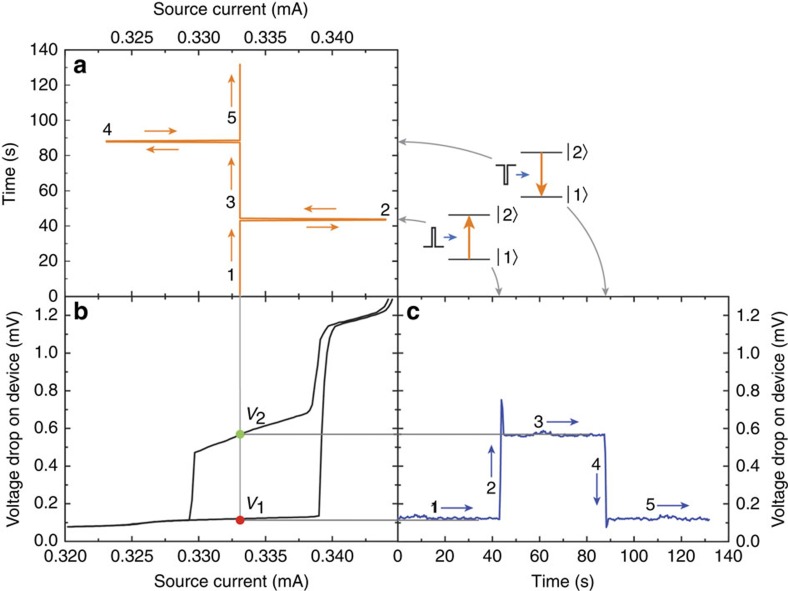
Controlled switching between two metastable states. (**a**) The current as a function of time *I*(*t*). (**b**) The *V*–*I* loop. (**c**) The voltage as a function of time *V*(*t*). The measurement starts along path →1 (see **a** and **c**) in state |1〉, at *V*_1_=0.12 mV and a bias current *I*_bias_=0.3331, mA. After 40 s, a current pulse perturbation Δ*I*=+0.0109, mA is applied (→2) for 0.1 s. The system rapidly switches to state |2〉, with *V*_2_=0.57 mV, at the same *I*_bias_(→3). Applying a 0.1-s dark pulse Δ*I*=−0.01 mA (→4) causes the system to return to the initial state |1〉 with voltage *V*_1_ at *I*_bias_ (→5).

Next, we test the idea of obtaining reproducible deterministic switching between dissipative states with an external perturbation by replacing the pink noise source with a current pulse generator. In Fig. 4 we show that by applying single-current pulses, we can switch between two distinct dissipative states. The external noise level was chosen so that the average frequency of switching is essentially zero at a fixed current (Fig. 3) and no unwanted switching occurs spontaneously. We start with a state *V*_1_ at a constant bias current *I*_0_=0.3331, mA. After increasing the current momentarily to 0.3440, mA, which is equivalent to applying a current pulse of magnitude Δ*I=*+0.0109, mA (duration 0.1 s), the system switches to higher dissipative state with *V*_2_, containing a higher number of PSCs than *V*_1_, and remains in this state thereafter.

Remarkably, down-switching can also be achieved: by applying a negative dark current spike (Δ*I*=−0.01 mA relative to the constant bias) for 0.1 s, the system returns to *V*_1_. The voltage ratio 
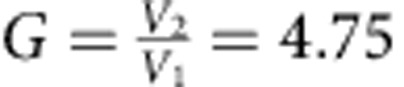
. Such deterministic switching—where the system changes state immediately after the pulse is applied—is obtained when the pulse amplitude exceeds the steady-state currents for the two pertinent states as shown in Fig. 4. Lower values of Δ*I* lead to probabilistic switching behaviour, similar to that observed after the application of noise (Fig. 3).

### Modelling

We can visualize the switching process between the metastable states shown in Fig. 2b in terms of solutions of TDGL equations^29^. In spite of the fact that TDGL theory is strictly valid only for the gapless case, it qualitatively describes current–voltage characteristics of narrow superconducting channels^28^ and predicts correct behaviour of the switching and retrapping currents^29^ in thin superconducting channels. The inherent nonlinearity of these equations lead to a remarkably faithful description of the observed switching behaviour. Including external control, they can be written as:


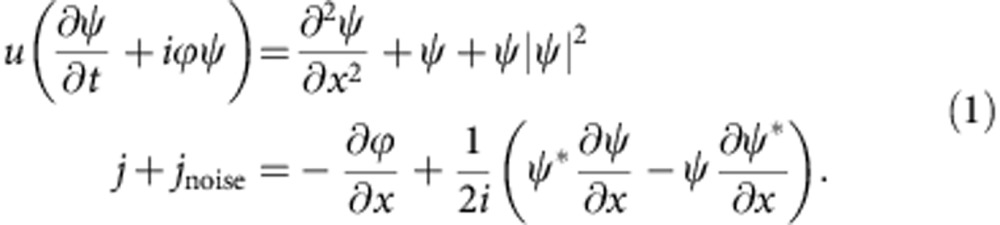


Here 
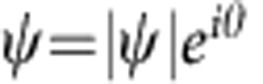
 is the dimensionless complex-order parameter, where *θ* is the phase of the order parameter, *ϕ* is the electrostatic potential, *u*=*τ*_GL_/*τ*_*θ*_ is a material-dependent parameter and *τ*_GL_ and *τ*_*θ*_ are the amplitude and phase relaxation times, respectively. The noise is introduced by adding a randomly fluctuating current density *j*_noise_=*rj*_0_. Here *r* denotes random numbers between −1 and 1, simulating white noise, and *j*_0_ is the magnitude of the noise current density, in units of critical current density *j*_c_. With appropriate conditions, the equations have two locally stable solutions with different number of PSCs for a fixed current *j* (for details please refer to Supplementary Note 3).

**Figure 5:**
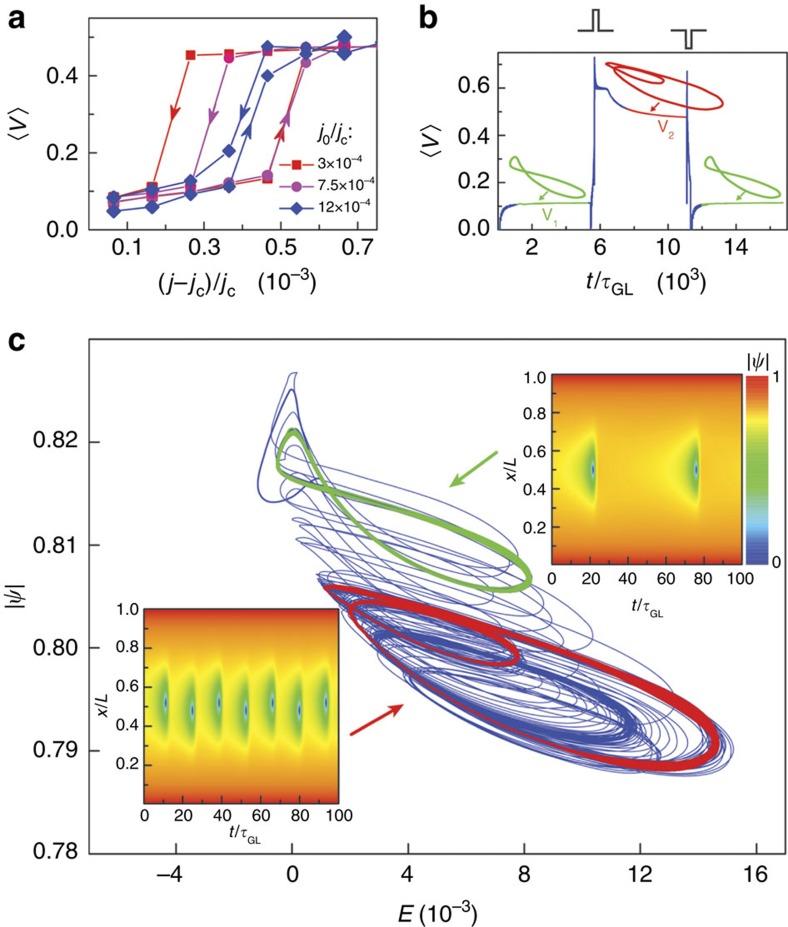
Modelled switching behaviour. (**a**) The average voltage 〈*V*〉 as a function of normalized current (*j*−*j*_c_)/*j*_c_ from model calculations. The different hysteresis loops are obtained with different noise amplitude *j*_0_. (**b**) The calculated average voltage 〈*V*〉 as a function of normalized time *t*/*τ*_GL_ shows the change in voltage as a result of switching with external +ve or –ve current pulses (shown). 
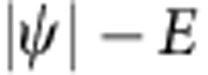
 parametric plots corresponding to the two calculated limit cycles for the two states |1〉 and |2〉 are shown in green and red. (**c**) The entire trajectory of the system though the entire switching process calculated for the midpoint of the wire, shown in the 
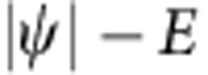
 plane. The initial and final state trajectories are emphasized in green and red, respectively. The spatio-temporal behaviour of the two states in the calculation correspond to one and two PSCs are shown in **d** and **e**, respectively. Note that before switching, the single PSC is pulsing in the centre of the wire (**d**), while after switching the two alternating PSCs are pulsing at a different frequency and are slightly displaced from the centre (**e**).

Figure 5a shows calculated average voltage *V* using equation (1) with appropriate boundary conditions applicable to our wire, for different noise amplitude *j*_0_. The switching occurs between states, which differ by one PSC, with *G*≃5. Note the excellent agreement between the measured and calculated effect of the external noise (Fig. 5a versus Fig. 2b): the loop and smoothness of the switching are reproduced, and *G* is also close to the experimental value. The magnitude of the simulated noise is also in reasonable agreement with the experimental values: the magnitude of the parameter in the model calculations is *j*_0_/*j*_c_=3∼12 × 10^−4^, very close to the experimental values *J*_r.m.s._/*J*_c_≅3.3∼11 × 10^−4^ given in Fig. 2b. Here the critical current (Fig. 2a) of the MoN nanowire *J*_c_≅3.3 × 10^−4^ A.

For pulse-triggered switching, the numerical solutions of equation (1) are shown in Fig. 5b. Initially, the system is in state with a voltage drop *V*_1_, and the system follows one limit cycle (green). When a short current pulse is supplied, the system will switch to a state containing two PSCs, the voltage drop will increase to *V*_2_, and the system will follow a different limit cycle (red). When an appropriate dark current pulse is applied, the system will revert to the initial state with one PSC. Comparing with Fig. 4c, we see that the modelling reproduces the experimentally observed deterministic switching behaviour.

In Fig. 5c we present a projection of the system trajectory through the switching process in a parametric plot 
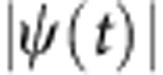
 versus *E*(*t*), where *E*(*t*)=−d*ϕ*(*t*)/d*x* is dimensionless electric field (Supplementary Note 3), calculated at the centre of the wire (at *x*=*L*/2, where *L* is the length of the wire). The spatio-temporal behaviour of the system before and after switching is shown in Fig. 5d,e, respectively. Before switching, a single PSC is observed exactly in the middle of the wire (*x*=*L*/2) pulsing at the Josephson frequency *ν*_J_=*e*〈*V*_1_〉/*h*. After switching, two alternating PSCs are observed, displaced from the centre, pulsing at a different frequency corresponding to 〈*V*_2_〉 shown in Fig. 5b.

## Discussion

The current-controlled switching may be useful as a low-temperature pulse-controlled resistive memory device. Considering that the nanowire itself acts as a nonlinear element there is no need for fabrication of delicate tunnel junctions commonly used in superconducting information-processing devices^27^. In effect, the δ-MoN nanowire may be viewed as a series of Josephson junctions, whose number is controlled by the passing current. Remarkably, the wire exhibits amplification in the sense that a tiny current perturbation Δ*I*=±0.01 mA causes a discrete and very large change of voltage across the device between *V*_1_ and *V*_2_, as the system switches from one limit cycle to another. The voltage gain *G*, defined by the ratio of voltages *V*_*j*_/*V*_*i*_ is determined by the number of PSCs in the wire, and can be altered by adjusting the bias current to select a particular loop, the maximum *G*=7.2 for this circuit (Fig. 2b). Here the two states were chosen for convenience, but Fig. 2 indicates the availability of multiple metastable states, which could be used to store data, which can be chosen simply by adjusting the external bias current.

## Methods

### Nanowire material preparation

δ-MoN nanowires were produced by transformation from Mo_6_S_*y*_I_*z*_ nanowires^31^ with diameters between 13 and 320 nm. The Mo_6_S_*y*_I_*z*_ nanowires used as templates were prepared from the elements in a single-zone furnace at *T*=1,040 °C (refs 31, 32) where *y* and *z* refer to the initial stoichiometry in the synthesis, within the range 8.2<*y*+*z*<10. The transformation of MoSI nanowires into δ_3_-MoN nanowires was carried out inside a tube furnace with a constant flow of argon and ammonia at *T*=825 °C (ref. 30). The structure and morphology of the MoN nanowires were characterized with high-resolution scanning electron microscopy, high-resolution transmission electron microscopy and atomic force microscopy. The cross-sectional high-resolution transmission electron microscopy image of a typical wire shows that it is composed of randomly oriented crystals of δ-MoN, with diameters between 10 and 50 nm.

### Electrical measurements

Wires with different diameters were dispersed in an ultrasonic bath in acetonitrile for 5 min at 80 kHz, and centrifuged at 385*g* to remove heavy agglomerates by sedimentation. The remaining dispersion was spray cast on a silica substrate and examined under scanning electron microscope. Focused ion beam-induced platinum deposition was made using beams with 30-kV acceleration voltage and current of 80–430 pA with a FEI Helios NanoLab 650. The thickness of the deposition is roughly 1 μm. The resistance measurements were done with the four-contact method using a Keithley 6221/Keithley 2182A set-up. Noise was introduced by attaching an external noise source as indicated in inset of Fig. 1b in the main text. The r.m.s. noise current *J*_r.m.s._ through the wire was varied between 0.044 and 0.36 μA, calculated from the measured voltages *V*_r.m.s._ indicated in Fig. 2b, using *J*_r.m.s._=*V*_r.m.s._/*R*_c_, where *R*_c_(∼6,340 Ω) is the total effective resistance of the circuit (two contacts, the nanowire and the current source).

## Additional information

**How to cite this article**: Buh, J. *et al*. Control of switching between metastable superconducting states in δ-MoN nanowires. *Nat. Commun.* 6:10250 doi: 10.1038/ncomms10250 (2015).

## Supplementary Material

Supplementary Information Supplementary Figures 1-3, Supplementary Notes 1-3 and Supplementary References
