# Supplementary material for: Control of switching between metastable superconducting states in δ-MoN nanowires
Source: Nat Commun. 2015 Dec 21;6:10250. doi: 10.1038/ncomms10250 (PMC4703890; doi:10.1038/ncomms10250)
Supplement: Supplementary Information — Supplementary Figures 1-3, Supplementary Notes 1-3 and Supplementary References [file ncomms10250-s1.pdf]

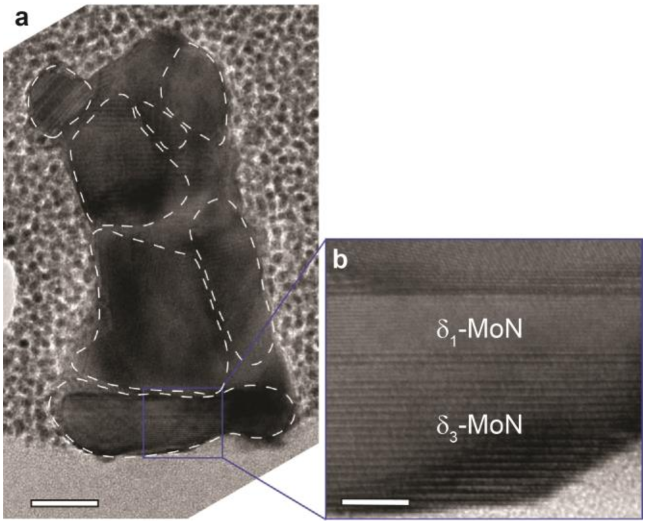

Supplementary Figure 1 | Transmission electron micrograph. a, A cross-sectional image of a single δ-MoN nanowire. The wire is laying on a SiO_2 substrate and is embedded in a Pt-C-Ga matrix. The edges of grains are approximately indicated by a dashed line (white marker corresponds to 20 nm). **b**, Enlargement is showing two different phases present in the cross-section. The lattice constant along the a axis is 286 pm for δ_1 phase and 573 pm for δ_3 phase according to Gannin *et al.*[1] The sample investigated in the main article had a high percentage of δ_3 phase due to optimised synthesis procedure (white marker corresponds to 5 nm).

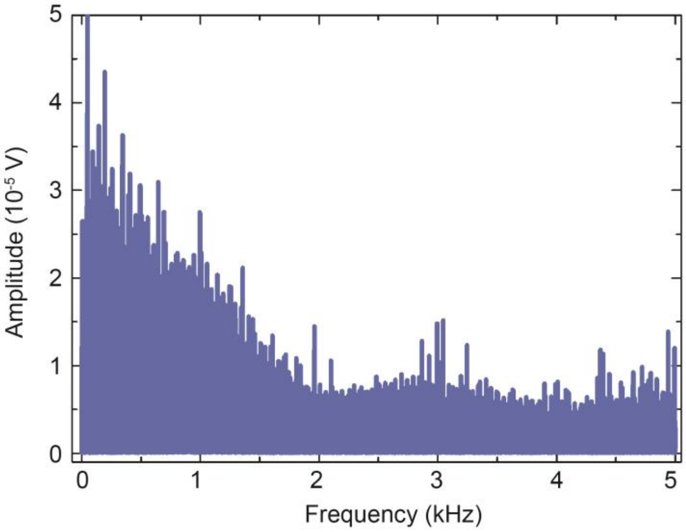

Supplementary Figure 2 | Spectrum of applied noise. Fourier spectrum of the root-mean-square noise in the experiment measured for $J_{RMS} = 0.22\ \mu$ A ($V_{RMS} = 1.38$ mV).

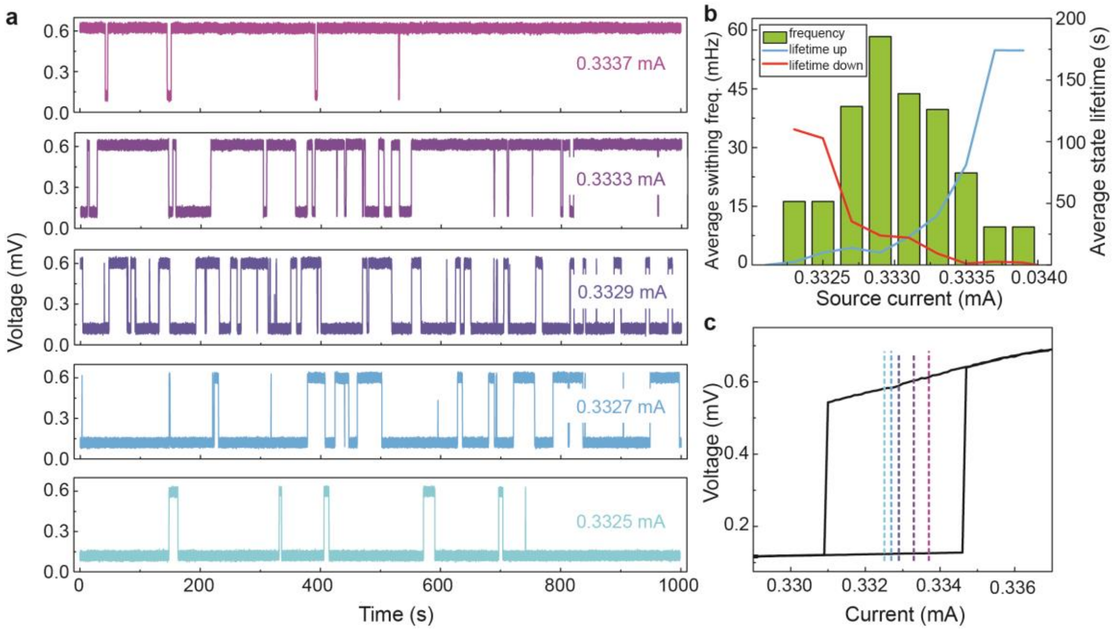

Supplementary Figure 3 | Current dependence of telegraph noise. a, Telegraph noise for different values of source current. **b**, The average switching frequency and the average lifetimes of the two states extracted from telegraph noise dynamics. **c**, The source currents from Supplementary Figure 3a marked relative to the current-voltage loop. For all the data on this figure the external noise has been held at 0.36 μA RMS.

Supplementary Note 1 - Sample characterization

We have prepared a TEM lamella of several MoN samples and one lamella of MoSI starting material with the focused ion beam site-selective procedure. The nanowire material is drop cast on a SiO_2 substrate and a protective layer of Pt is deposited over a selected nanowires. A lamella containing several cross-sections is extracted. Results of the subsequent TEM study for MoN can be seen in Supplementary Figure 1. The starting MoSI material is highly crystalline. After transformation TEM cross-section of a δ-MoN nanowire with synthesis parameters $T = 825$ °C, $t = 4$ h, $\varphi(\mathrm{HN_3}) = 15$ ml/min shows a polycrystalline material with the average grain size ≈ 20 nm. At higher magnifications we

can see two different phases present in the cross-section. The lattice constant along the a axis is 286 pm for δ_1 phase and 573 pm for δ_3 phase according to Gannin *et al.* [1].

Supplementary Note 2 - Noise characteristics and current dependence of the telegraph noise.

The external noise was approximated by an antenna gathering ambient electrical noise on the outside of the cryostat connected directly to the sample contact. The Fourier spectrum of the source is shown in Supplementary Figure 2 for $J_{\mathrm{RMS}} = 0.22\ \mu$ A ($\mathrm{V_{RMS}} =$ 1.38 mV) amplitude noise. The spectrum was found to be stable, day to day variations in the spectrum were imperceptible (including the spikes), and did not give rise to any detectable qualitative differences in the device switching behavior. The noise spectrum is approximately flat above 1.6 kHz, but increases below this frequency to approx. 3 times the high frequency value at 10 Hz.

Although the cycling behavior in Figure 2 appears qualitatively reproducible, the switching does not occur exactly at the same current in repeated cycles. Instead, it is governed by a statistical process, which is controlled by the noise level. In Supplementary Figure 3a we show the temporal behavior of the switching process in several sub-section of the hysteresis loop, keeping a constant noise level $J_{\mathrm{RMS}} = 0.36\ \mu\mathrm{A}$. At low currents, the system remains predominantly in the bottom state with low average switching frequency. Upon increasing I, the number of transitions per unit time first increases while the system is predominantly in bottom state. As the current is further increased, the switching rate is maximum, and then decreases again, as the system is predominantly in top state. The rate and the average life-times of the both states are shown in Supplementary figure 3b. The currents supplied to the wire in Supplementary Figure 3a are marked relative to the hysteresis loop in Supplementary Figure 3c. The range of currents at which the switching

occurs δI, is much smaller than the width of the hysteresis loops shown in Figure 2b in the main text.

Supplementary Note 3 - Calculation details

The TDGL Equations (1) in the main text are written in dimensionless units. Following refs. 2 and 3, we measure the distance x and time t in units of the coherence length ξ and phase relaxation time $\tau_{\theta} = 4\pi\lambda^2\sigma_{\mathrm{n}}/c^2$ respectively. λ is the penetration depth, σ_{n} is the normal state conductivity, and c is the speed of light. The electrostatic potential φ is measured in the units of $\phi_0/(2\pi c\tau_{\theta})$, where $\phi_0 = \pi\hbar c/e$ is the flux quantum, e is the electronic charge and $\hbar$ is the reduced Planck's constant and the electric field is measured in units of $\phi_0/(2\pi c\tau_{\theta}\xi)$. We measure the current in units of Ginzburg-Landau depairing current for an infinite channel $j_{\mathrm{c}} = 0.385\ \phi_0 c/(8\pi^2\lambda^2\xi)$. The penetration depth of electric field l_{E} is an additional parameter, which characterizes the nonequilibrium superconductors. From Equations (1) in the main text it follows that $l_{\mathrm{E}} = \xi/u^{1/2}$. Therefore, the parameter u characterizes the penetration of the electric field in the nonequilibrium superconductors[2]. In order to reproduce the hysteresis loop obtained in ref. 3 we set the parameter $u = \tau_{GL}/\tau_{\theta} = 1$ and the length of the channel $L/\xi = 23.56$.

The time integration of the TDGL equations has been done by Euler method. The spatial derivatives have been evaluated using the finite difference scheme of the fourth order. We have studied the TDGL equations in the constant current regime. Since we know the value of the total current, we can estimate the electrostatic potential φ and, therefore, voltage V.

The boundary conditions were the following:

$$\rho(-L/2) = \rho(L/2) = 1, \qquad \frac{\partial\varphi(-L/2)}{\partial x} = \frac{\partial\varphi(L/2)}{\partial x} = 0, \tag{1}$$

and the absence of the electric field at the edges of the channel determined the phase gradient:

$$\frac{\partial\theta(-L/2)}{\partial x} = \frac{\partial\theta(L/2)}{\partial x} = j. \tag{2}$$

Supplementary references

1. Ganin, A. Y., Kienle, L. & Vajenine, G. V. Synthesis and characterisation of hexagonal molybdenum nitrides. *J. Solid State Chem.* **179,** 2339–2348 (2006).
2. Kopnin, N. B. *Theory of Nonequilibrium Superconductivity*. (Clarendon Press, 2001).
3. Baranov, V. V., Balanov, A. G. & Kabanov, V. V. Dynamics of resistive state in thin superconducting channels. *Phys. Rev. B* **87,** 174516 (2013).
